# Bioprocessed poultry by-product meals on growth, gut health and fatty acid synthesis of juvenile barramundi, *Lates calcarifer* (Bloch)

**DOI:** 10.1371/journal.pone.0215025

**Published:** 2019-04-09

**Authors:** Muhammad A. B. Siddik, Patience Chungu, Ravi Fotedar, Janet Howieson

**Affiliations:** 1 School of Molecular and Life Sciences, Curtin University, Bentley, WA, Australia; 2 Department of Fisheries Biology and Genetics, Patuakhali Science and Technology University, Patuakhali, Bangladesh; 3 National Aquaculture Research and Development Centre, Department of Fisheries, Kitwe, Zambia; Universiti Sains Malaysia, MALAYSIA

## Abstract

Poultry by-product meal (PBM) has been utilised as a substitute of fishmeal (FM) in many aquaculture species. However, little information is known regarding the use of bioprocessed PBM (BPBM) in aquaculture production. This study was undertaken to investigate whether replacing FM with BPBM improved growth performance, gut morphology and fatty acid synthesis of juvenile barramundi, *Lates calcarifer*. The PBM was bioprocessed by baker yeast, *Saccharomyces cerevisae* and *Lactobacillus casei*. The BPBM was used to replace FM at 75% and 100% (75BPBM and 100BPBM) contrasting against unprocessed PBM (75PBM and 100PBM) at the same levels and FM based diets as the control. Juvenile barramundi with a mean initial weight of 3.78±0.16 g were stocked at a density of 20 fish per tank. After the 42 days of study, the final weight, specific growth rate and feed conversion ratios of fish fed 75PBM and 75BPBM were not significantly different from the control. However, 100% supplementation diets of 100PBM and 100BPBM resulted in reduced performance in all growth and feed variables except total feed intake and survival. The hind gut microvillus density was significantly higher (P<0.05) in fish fed 75BPBM, whereas the microvillus diameter remained unaffected with the other experimental diets when compared to the control. A reduction in eicosapentaenoic (EPA) and docosahexaenoic (DHA) acids of fish muscles led to a lower Σn−3/Σn−6 ratio in all dietary groups when compared to the control. The percentage of Σn-3 PUFAs decreased in 100% FM replacement diets of 100PBM and 100BPBM, while Σn−6 PUFAs increased when both bioprocessed and unprocessed PBM protein was increased in the diets. Fish fed bioprocessed diets had higher fatty acid hypocholesterolemic/hypercholesterolemic ratios (HH), indicating improved suitability for human consumption.

## Introduction

Fishmeal (FM) is one of the best dietary sources of protein in aqua-diets due to a high protein content, balanced amino acid profile, elevated omega-3 polyunsaturated fatty acid, high protein digestibility, and excellent palatability properties [1, 2]. Unfortunately, a shortage of raw materials due to the progressive depletion of fish stocks, coupled with soaring demand and economic issues has impacted the sustainable development of aquaculture production [3]. Therefore, there is a growing interest in the aquaculture production sector to find alternative protein sources of FM that are locally available, sustainable and cost effective. Over the last decades, there has been an unprecedented number of studies conducted on plant protein sources as possible alternative feed ingredients for aquatic species [4]. The major limitation in plant feedstuffs is that they lack essential long chain omega 3 fatty acids and are naturally rich in carbohydrate which is poorly metabolized by carnivorous fish [5, 6]. The potential use of rendered animal by-products, including PBM, meat and bone meal, feather meal and fish offal meal for aquatic feeds is not new, with such nutritional potential recognized at the very inception of fish farming. Over the years, significant research has been conducted on PBM due to its high protein content and favourable amino acid profile [7, 8]. PBM is also readily available and often cheaper due to the low demand from other sectors such as food and pharmaceutical companies [4, 9].

Many studies have investigated the efficacy of PBM on various fish species and success was generally only reported when PBM partially replaced FM in the diets [10, 11]. Moreover, the utilization of high levels of PBM to replace FM has resulted in depressed growth performance of fish [12]. Several studies have confirmed that higher inclusion levels of PBM in fish diets, also resulted in declines of methionine and lysine levels [13, 14], which are the major impediments to incorporating substantial levels of PBM into the diets of carnivorous fish. Another major concern with using PBM in aqua diets is its poor digestibility giving variable nutritive value [15]. Simon, Salini [15] stated that freshness of raw materials and their processing conditions are the main factors determining the quality of the final product. The amount of heat and moisture applied to the material during the rendering process has major implications to the feed quality and utilisation of the finished meal [16]. Like other terrestrial animal by-products, PBM is also deficient in adequate proportions of n-3 polyunsaturated fatty acids such as eicosapentaenoic acid (EPA; 20:5n-3) and docosahexaenoic acid (DHA; 22:6n-3) [17], potentially lowering the nutritional quality of fish meat intended for human consumption.

A possible strategy to circumvent these shortcomings is using a bioprocessing or fermentation technique - an environmentally suitable and cost effective method used to overcome many of the inherent problems of animal by-product protein [18–21] to make it suitable for inclusion in fish diet formulations [22–24]. Fermentation breaks down carbohydrates into a form that makes the innate energy and protein digestible [25], whilst improving the nutritional quality of animal by-products by producing low-molecular-weight compounds that potentially enhance mineral absorption [2, 26], amino acids profile [27], and reduce anti-nutritional factors [25, 28]. Fagbenro and Jauncey [29] found that feeds made from fermented products tend to have higher stability in water, thereby allowing more time for fish to ingest the feed and maximise nutrient intake. Furthermore, fermented feeds are characterized by high amounts of lactic acid bacteria [30] which can proliferate in the gut and produce high concentrations of beneficial lactic acid, as well as several volatile fatty acids including acetic acid, butyric acid, and propionic acid [31].

Barramundi, *Lates calcarifer* (Bloch), a common species in the Indo-Pacific region and Australia, is gaining much attention from farmers and researchers [2]. Due to its wide range of salinity tolerance, adaption capacity in versatile farming systems and highly appreciated meat, barramundi is progressively becoming a major commercial species in aquaculture [15]. Thus, it is necessary to establish a cost-effective growth promoting diet for a continuing and feasible barramundi farming industry. To the authors’ knowledge, growth studies looking at the potential of PBM to replace FM with emphasis on gut health and fatty acid synthesis, remains unassessed. The present study therefore aimed to evaluate the effects of graded levels of bioprocessed and unprocessed PBM on growth performance, nutritional composition, gut and liver health, and fatty acid synthesis in juvenile barramundi.

## Materials and methods

### Ethics

This experiment was conducted under the guidance of the Care and Use of Laboratory Animals in Australia. The procedures and protocols of treating fish in this study were approved by the Animal Ethics Committee of the Curtin University, Australia (Approval Number: AEC_2015_41). In short, fish were starved for 24 h prior to being anaesthetised (AQUI-S, 8 mgL^−1^), weighed and taking blood samples. After completion of the trial, remaining fish were euthanized according to CARL SOP Euthanasia of Fish, using AQUI-S.

### Experimental diets

The formulation and nutrient composition of the experimental diets are presented in Table 1. All feed ingredients for this study were procured from Specialty Feeds, Glen Forrest Stockfeeders, Perth, Western Australia. The PBM was sieved through a 0.5mm size mesh sieve and was used as the raw material for bioprocessing. The fermentation of PBM was completed following a technique described in our earlier study [32]. In short, PBM was weighed and Baker’s yeast, *Saccharomyces cereviceae* (Instant dried yeast, Lowan) was added at 10% and *Lactobacillus casei* in the form of skim milk product (Yakult, cell density of 3 × 10^6^ CFU ml^−1^) was added at 5% of the weight of PBM. Distilled water was then added at approximately 70% of the weight of the total meal mixture and all ingredients were thoroughly mixed in a food mixer. The mixture was then placed in an Erlenmeyer flask covered with aluminium foil and incubated at 30°C for 4 days. The fermented product was dried in an oven at 60°C for 24 h and used as a feed ingredient. Five isonitrogenous and isocalorific diets having 48.0% crude protein and 20.0 MJ kg^−1^ gross energy were formulated based on bioprocessed and unprocessed PBM to replace FM at 75% (75PBM and 75BPBM) and at 100% (100PBM and 100BPBM). The control diet was formulated based on FM as the main protein source. The experimental diets were prepared based on the standard protocol of Curtin Aquatic Research Laboratories (CARL) and met the nutrient requirements of juvenile barramundi according to NRC [33]. The amino acids (AAs) and fatty acids (FAs) composition of the experimental diets and tested unprocessed and bioprocessed PBM are presented in Table 2 and Table 3, respectively.

**Table 1:** Formulations and nutrient composition of experimental diets fed on juvenile barramundi.

*Test Ingredients*^*1*^	Experimental diets (g kg^−1^ DM)				
	Control	75PBM	75BPBM	100PBM	100BPBM
Fishmeal^2^	610.0	152.50	160.0	-	-
PBM^3^	-	429.0	-	575.0	-
BPBM^4^	-	-	439.0	-	595.0
Wheat flour	266.0	290.0	272.50	300.o	280.0
Wheat starch	20.0	20.0	20.0	20.0	20.0
Fish oil	30.0	30.0	30.0	30.0	30.0
Calcium carbonate	20.0	20.0	20.0	20.0	20.0
Salt (NaCl)	20.0	20.0	20.0	20.0	20.0
Vitamin premix	10.0	10.0	10.0	10.0	10.0
Casein	63.0	65.0	65.0	65.0	65.0
Cellulose	60.0	85.0	85.0	50.0	50.0
*Nutrient composition (% dry weight)*					
Crude protein	48.91	48.33	48.73	48.31	48.04
Crude Lipid	9.99	10.93	10.19	11.29	10.49
Ash	12.87	9.18	9.55	8.31	8.35
Moisture	17.93	14.20	11.86	13.98	11.50
NFE^5^	28.23	31.56	31.53	32.09	33.12
Gross energy (MJ kg^-1^)	19.84	20.62	19.97	20.35	20.21

^1^Supplied by Specialty Feeds, Perth, Australia.

^2^Fishmeal: 64.0% crude protein, 10.76% crude lipid and 19.12% ash.

^3^PBM (poultry by-product meal): 67.13% crude protein, 13.52% crude lipid and 13.34% ash.

^4^BPBM (Bioprocessed poultry by-product meal): 66.98% crude protein, 11.70% lipid and 14.68% ash.

^5^Nitrogen free extracts (NFE) = dry matter—(crude lipid + crude ash+ crude protein).

**Table 2:** The amino acid composition (g/100g) of the five experimental diets and tested unprocessed and bioprocessed PBM.

	Experimental diets					PBM	BPBM
	Control	75PBM	75BPBM	100PBM	100BPBM		
***Essential amino acids***							
Phenylalanine	2.05	2.02	2.02	2.19	2.02	2.65	2.65
Glutamic acid	7.70	8.42	8.54	8.67	8.54	9.55	9.84
Leucine	3.88	3.71	3.75	4.00	3.75	5.00	4.97
Lysine	3.25	2.83	2.95	3.01	2.95	4.17	4.21
Methionine	1.30	1.06	1.02	1.21	1.02	1.46	1.43
Isoleucine	2.12	1.99	2.06	2.20	2.06	2.67	2.66
Histidine	1.35	1.05	1.00	1.22	1.00	1.47	1.39
Threonine	2.21	2.00	1.97	2.20	1.97	2.81	2.86
Valine	2.41	2.34	2.41	2.55	2.41	3.08	3.11
***Non-essential amino acid***							
Arginine	2.92	3.49	3.18	3.39	3.18	5.43	5.24
Alanine	2.88	3.01	3.15	3.23	3.15	4.62	4.89
Taurine	0.11	0.19	0.12	0.21	0.12	0.31	0.31
Tyrosine	1.71	1.70	1.67	1.83	1.67	2.15	2.17
Glycine	3.11	4.19	4.14	4.04	4.14	6.53	6.79
Aspartic acid	4.43	3.98	4.15	4.34	4.15	5.95	6.03
Cysteine	0.44	0.50	0.53	0.57	0.53	0.66	0.69
Serine	2.32	2.34	2.26	2.48	2.26	3.01	3.08
Proline	3.48	5.72	4.37	5.67	4.37	5.63	5.94

**Table 3:** The fatty acid composition (% of total fatty acids) and total fatty acid content (mg/g of lipid) of the five experimental diets and tested unprocessed and bioprocessed PBM.

Fatty acids	Experimental diets					PBM	BPBM
	Control	75PBM	75BPBM	100PBM	100BPBM		
C12:0	0.06	0.07	0.08	0.08	0.10	0.09	0.08
C13:0	0.04	0.09	0.02	0.03	0.03	0.02	-
C14:0	2.85	1.92	1.73	1.65	1.66	0.97	0.85
C14:1n-5	0.04	0.12	0.12	0.15	0.14	0.21	0.18
C15:0	0.72	0.35	0.34	0.21	0.23	0.20	0.18
C15:1	0.11	0.07	0.06	0.01	0.01	0.03	0.03
C16:0	17.76	18.61	19.07	18.80	19.17	14.13	21.58
C16:1n-7	3.37	4.23	4.64	4.52	5.04	5.36	5.65
C17:0	1.09	0.51	0.53	0.31	0.34	0.38	0.36
C17:1	0.58	0.36	0.35	0.30	0.32	0.24	0.24
C18:0	5.71	6.21	6.58	6.35	6.71	9.38	8.53
C18:1cis or trans	16.43	31.92	32.16	36.97	37.31	49.39	42.25
C18:2 trans 9	0.13	0.09	0.10	0.10	0.08	0.15	0.12
C18:2n-6	5.61	13.47	13.28	15.62	14.59	13.68	13.72
C18:3n6	0.15	0.16	0.16	0.15	0.13	0.12	0.13
C18:3n3	1.33	2.32	2.15	2.55	2.29	1.6	1.85
C18:4n-3	0.98	0.77	0.62	0.68	0.67	0.06	0.06
C20:0	0.29	0.21	0.21	0.19	0.19	0.18	0.17
C20:1	2.31	2.01	1.7	1.95	1.99	0.67	0.59
C20:2	0.35	0.31	0.27	0.30	0.29	0.16	0.16
C20:3n-3	0.22	0.14	0.12	0.12	0.12	-	-
C20:3n-6	0.18	0.25	0.24	0.26	0.24	0.24	0.25
C20:4n-6	1.74	1.46	1.51	1.34	1.13	1.21	1.31
C20:5n-3 (EPA)	6.65	3.32	2.99	2.27	2.23	0.13	0.19
C21:0	0.12	0.09	0.08	0.07	0.07	0.08	0.07
C22:0	0.02	0.01	-	-	-	0.13	0.10
C22:1n-9	0.29	0.25	0.20	0.23	0.24	0.12	0.06
C22:2	0.03	0.03	0.02	0.02	0.02	-	-
C22:4n-6	1.96	0.54	0.60	0.12	0.11	-	0.06
C22:5n-3	1.92	1.3	1.13	1.1	1.04	0.41	0.35
C22:6n-3 (DHA)	26.31	8.48	8.63	2.99	2.94	0.24	0.45
C23:0	0.07	0.03	0.03	0.38	0.38	0.39	0.38
C24:0	0.16	0.07	0.07	0.03	0.03	-	0.03
C24:1	0.42	0.23	0.20	0.15	0.15	0.02	0.03
ΣSFA	28.89	28.17	28.74	28.1	28.91	25.95	32.33
ΣMUFA	23.55	39.19	39.43	44.28	45.2	56.04	49.03
ΣPUFA	47.56	32.64	31.82	27.62	25.88	18.0	18.65
Σn-3	37.41	16.33	15.64	9.71	9.29	2.44	2.90
Σn-6	9.64	15.88	15.79	17.49	16.2	15.25	15.47
Σn-3/ Σ n-6	3.88	1.01	0.99	0.56	0.57	0.16	0.19
Total Fatty Acids (mg/g)	836.8	891.8	859.6	941.2	898.6	1019.7	1054.7

EPA: Eicosapentaenoic acid, DHA: docosahexaenoic acid, ΣSFA, sum of saturated fatty acids; ΣMUFA, sum of monounsaturated fatty acids; ΣPUFA, sum of polyunsaturated fatty acids; Σn-3 PUFA, sum of omega-3 polyunsaturated fatty acids; Σn-6 PUFA, sum of omega-6 polyunsaturated fatty acids; IA, index of atherogenicity; IT, index of thrombogenicity and HH, (hypocholesterolemic/hypercholesterolemic ratio, -, not detected.

### Fish, experimental conditions and feeding

Juvenile barramundi were sourced from the Australian Centre for Applied Aquaculture Research, Fremantle, Australia. Fish were acclimatized to the laboratory conditions for 14 days. During the acclimation period, fish were fed twice daily with a basal formulated diet (48.0% crude protein and 20.0 MJ kg^-1^ dietary gross energy). Following acclimation, a total of 300 uniformly sized juvenile barramundi (mean initial weight of 3.78±0.16 g fish^-1^) were randomly distributed into fifteen tanks (300-L water capacity) at a stocking density of 20 fish per tank. Each tank was connected with an aerator, water heater and external bio-filter (Fluvial 406, Hagen, Italy) exchanging water at a rate of 10 L min^−1^. Fish were raised in saltwater medium with a salinity range of 31.0 to 34.0 ppt. The water quality parameters such as temperature, salinity, dissolved oxygen, ammonia and nitrite were monitored daily and were within the suitable range for fish culture in a recirculating aquaculture system [34]. Fish were kept at a 12:12 light:dark cycle. Throughout the experimental period of 42 days, fish were fed to satiation the respective diets three times a day at 0800, 1200 and 1700 h. About 1.5 h after feeding, uneaten feed was removed carefully by siphoning, transferred to aluminium cups, and dried to a constant weight in order to calculate the feed conversion ratio. The growth performance, feed utilization and body indices parameters of juvenile barramundi were calculated using the equations describe previously [32].

### Intestinal microvilli morphology

The potential effects of bioprocessed and unprocessed PBM on the ultrastructure of gut morphology were investigated by scanning electron microscopy (SEM). The SEM images (magnification × 30,000) were analysed to assess the microvilli density on the surface of enterocytes standardised to the 1 μm^2^ region [35]. The distal intestinal samples of three fish from each treatment (randomly selected one fish from each replicated tank) were considered for microscopic examination. For accuracy, at least 100 independent measurements were taken from the SEM images per treatment. The details of SEM sample preparation and data calculation were described previously in Ran, Huang [36].

### Histopathology

After being fed for 42 days, three randomly selected fish from each treatment were dissected for liver histological analysis. Liver samples were dehydrated in ethanol, equilibrated in xylene and embedded in paraffin wax following standard histological techniques. The sections of approximately 5 mm in size were cut and stained with Hematoxylin-Eosin (H&E) stain, for histological examination under a light microscope (BX40F4, Olympus, Tokyo, Japan).

### Biochemical analysis

Fish muscles and experimental diets were analysed for proximate composition based on the Association of Official Analytical Chemists procedures [37]. After termination of the feeding trial, three fish from each tank (9 samples per dietary treatment) were randomly selected, collected muscle tissues, freeze dried and grounded for biochemical analysis. The dry matter was determined by oven drying to constant weight at 105°C; crude ash by combustion at 550°C; crude protein content (N × 6.25) by the Kjeldahl digestion method; crude lipid content by the Soxhlet technique; and gross energy content of diets by an IKA oxygen bomb calorimeter (Heitersheim, Germany). The fatty acid composition of experimental diets and fish samples was performed following the method described by O'Fallon, Busboom [38].

### Indices of lipid quality

The lipid quality of fish fed bioprocessed and non-processed PBM was investigated by analysing the fatty acid profile of fish with three important indicators, namely, index of atherogenicity (IA), index of thrombogenicity (IT), and the fatty acids hypocholesterolemic/hypercholesterolemic ratio (HH) as follows:

(1) IA = [(C12:0 + (4 × C14:0) + C16:0)]/[(ΣMUFA + Σ(n-6) + Σ(n-3)] [39]

(2) IT = (C14:0 + C16:0 + C18:0)/[(0.5×ΣMUFA + 0.5×Σ(n-6) + 3×Σ(n-3) + Σn-3/Σn-6] [39]

(3) HH = (C18:1cis9 + C18:2n6 + C20:4n6 + C18:3n3 + C20:5n3 + C22:5n3 + C22:6n3)/(C14:0 + 16:0) [40]

### Statistical analysis

Results of growth performance, nutritional composition and fatty acid profiles are compared by one way analyses of variance (ANOVA), using SPSS for Windows version 25, IBM Curtin University, Australia. Normality of the data was previously assessed by a Shapiro-Wilk’s test and homogeneity of variances was verified using the Levene's test. If ANOVA of values were significant (P<0.05), Tukey or Duncan's post hoc tests for multiple pairwise comparisons was then applied. The principal component analysis (PCA) was applied to correlate variables to which fatty acid in fish muscles differed between dietary treatments of bioprocessed and unprocessed PBM using PAST 3.15 software.

## Results

### Growth and body indexes

The results of growth performance, feed utilization, body indices and gut micro-morphological indices of fish are shown in Table 4. A significant (P<0.05) decrease in final body weight (FBW), weight gain (WG) and specific growth rate (SGR) as well as increased feed conversion ratio (FCR), were found in fish fed 100PBM and 100BPBM when compared to the control, and the 75PBM and 75BPBM. The study also indicated that bioprocessing of PBM has no positive influence on the growth performance over unprocessed PBM. Total feed intake and survival was identical among the treatments. The percentage of hepatosomatic index (HSI) was significantly lower in the control and 75% replacement groups of 75PBM and 75BPBM when compared to the total replacement groups of 100PBM and 100BPBM, while the viscerasomatic index (VSI) and condition factor (CF) were not influenced in fish fed bioprocessed and unprocessed PBM at varying levels when compared to the control.

**Table 4:** Growth performance, feed utilization and gut micro-morphological indices of juvenile barramundi fed with different levels of bioprocessed and unprocessed PBM.

	Experimental diets				
	Control	75PBM	75BPBM	100PBM	100BPBM
*Growth performance*					
FBW (g/fish)	32.36 ± 1.94^a^	32.72 ± 1.39^a^	33.73 ± 1.28^a^	26.56± 2.04^b^	26.32 ± 2.43^b^
WG (g/fish)	28.81 ± 1.94^a^	28.96 ± 1.22^a^	29.84 ± 1.21^a^	22.58± 2.45^b^	22.59 ± 1.59^b^
SGR (%/day)	5.27 ± 0.15^a^	5.15 ± 0.07^a^	5.14 ± 0.05^a^	4.52 ± 0.19^b^	4.63 ± 0.25^b^
Survival (%)	88.33± 4.41	91.67± 1.67	86.67± 1.67	90.0± 2.89	81.67± 3.33
*Feed utilization*					
TFI (g/fish)	36.56±2.45	34.30± 0.35	34.17 ±1.58	31.20 ± 2.03	33.36 ± 2.05
FCR	1.27 ± 0.03^a^	1.19 ± 0.09^a^	1.15 ±0.07^a^	1.41 ± 0.15^b^	1.50 ±0.10^b^
*Body indexes*					
CF (g/cm^3^)	1.26±0.05	1.22±0.05	1.24±0.04	1.18±0.04	1.16±0.06
HSI (%)	1.56±0.12^bc^	1.77±0.07^b^	1.49±0.10^c^	1.99±0.07^a^	1.83±0.05^a^
VSI (%)	9.15±0.14	10.25±0.97	9.62±0.19	9.77±0.78	9.35±0.29
*Gut micro-morphology*					
Microvilli density (count/μm^2^)	125.27±2.01^b^	125.80±1.71^b^	134.80±1.75^a^	116.73±1.75c	112.20±1.85^c^
Microvilli diameter (μm)	0.11±0.00	0.12±0.00	0.11±0.00	0.10±0.00	0.11±0.00

Values are mean ± SE of three replicate tanks. Values in the same row with different superscript letters (a,b,c) are significantly different based on Duncan’s multiple range test (One-way ANOVA, P<0.05).

Final body weight (FBW, g)

$Weight\;gain\;(WG,g/fish)=\left[\frac{mean\;final\;body\;weight–mean\;initial\;body\;weight}{mean\;initial\;body\;weight}\right]$

$Specific\;growth\;rate\;(SGR,\%/day)=\left[\frac{ln\;(final\;body\;weight)-ln\;(pooled\;initial\;body\;weight)}{days}\right]\times 100$

$Feed\;intake\;(TFI,g)=\left[\frac{dry\;feed\;consumed}{fish\;number}\right]$

$Feed\;conversion\;ratio\;(FCR)=\left[\frac{dry\;feed\;fed}{wet\;weight\;gain}\right]$

$Survival\;(\%)=\left[\frac{number\;of\;final\;fish-number\;of\;initial\;fish}{number\;of\;initial\;fish}\right]\times 100$

$Condition\;factor\;(CF,g/cm3)=\left[\frac{final\;body\;weight}{length}\right]\times 100$

$Hepatosomatic\;index\;(HSI,\%)=\left[\frac{liver\;weight}{body\;weight}\right]\times 100$

$Viscerosomatic\;index\;(VSI,\%)=\left[\frac{visceral\;weight}{body\;weight}\right]\times 100$

### Gut microvilli morphology

**Fig 1:**
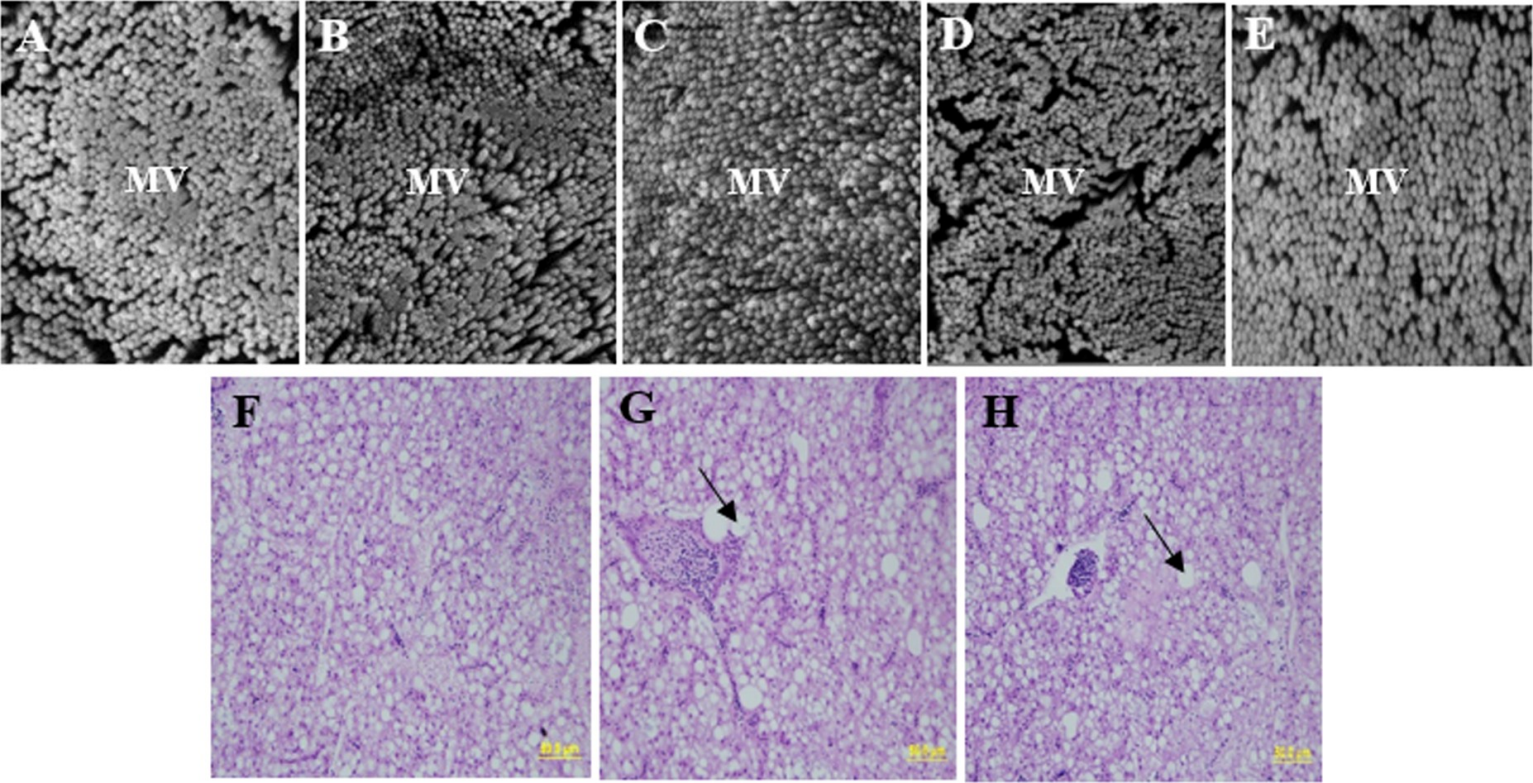
Gut microvilli and liver histological structure of juvenile barramundi. High magnification (x 30,000) electron micrographs showing microvilli in the distal gut of juvenile barramundi fed five experimental diets control, 75PBM, 75BPBM, 100PBM and 100BPBM (Panel A-E). MV = microvilli, Scale bar = 2 μm, microvilli density count per μm^2^. Histological structure of the liver of juvenile barramundi fed control, 100PBM and 100BPBM diets (Panel F-H). Black arrow in liver micrographs indicates large vacuoles in hepatic cells (H&E staining, 400x magnification, scale bar = 50μm).

SEM analyses showed that fish fed the 100% FM replacement diets of 100PBM and 100BPBM had lower gut microvilli density when compared to the control and the other two test diets. Significantly (P<0.05), the highest and the lowest microvilli density were found in fish fed 75BPBM and 100PBM, respectively, when compared to the control. However, the gut microvilli diameter of juvenile barramundi was not influenced, either by bioprocessed nor unprocessed PBM in the diets (Fig 1).

### Liver histopathology

No histological alterations were observed in fish fed the control, and 75% replacement diets of 75PBM and 75BPBM. These treatments were characterized by normal structure with balanced hexagonal hepatocytes and rare cytoplasmic vacuolization. However, hepatocytes from fish fed the 100% replacement diets of 100PBM and 100BPBM showed irregular arrangement of liver samples and cytoplasmic vacuolization with lipid deposition (Fig 1).

### Proximate composition and amino acid profile

The muscles proximate composition and amino acid profile of the juvenile barramundi fed the experimental diets are shown in Table 5. There were no significant (P>0.05) differences in moisture, protein, lipid and ash content of juvenile barramundi among the different dietary groups. The percentages of amino acids (AAs) such as Phenylalanine, Taurine, Cysteine and Serine content in muscles of fish fed PBM diets were similar to the control, whereas the remaining AAs were significantly different. The Methionine and Lysine content of fish fed control was significantly higher than the unprocessed diets but was similar to the fish fed bioprocessed diets (Table 5).

**Table 5:** Proximate composition (% wet weight basis) and amino acid profile (g/100g) of muscle tissue in juvenile barramundi at the end of feeding trial for 42 days.

	Experimental diets				
	Control	75PBM	75BPBM	100PBM	100BPBM
***Proximate composition***					
Moisture	73.81 ± 0.64	73.38 ± 0.72	74.47 ± 0.72	72.9 ± 0.78	74.28 ± 1.01
Crude protein	15.03 ± 0.34	15.12 ± 0.48	14.65 ± 0.47	14.57 ± 0.46	14.12 ± 0.30
Crude lipid	1.77 ± 0.22	1.87 ± 0.12	1.79 ± 0.07	2.07 ± 0.21	1.84±0.14
Ash	4.78±0.26	4.69±0.09	4.13 ± 0.07	4.74 ± 0.17	4.71 ± 0.06
***Essential amino acid***					
Phenylalanine	4.02±0.09	3.27±0.23	3.54±0.24	3.21±0.13	3.60±0.14
Glutamic acid	14.67±0.34^a^	12.17±0.13^c^	13.47±0.17^b^	11.96±0.19^c^	13.47±0.12^b^
Leucine	7.56±0.09^a^	6.32±0.10^c^	6.94±0.09^b^	6.13±0.10^c^	6.96±0.15^b^
Lysine	8.19±0.09^a^	7.05±0.15^b^	7.94±0.12^a^	6.96±0.37^b^	7.95±0.23^a^
Methionine	2.73±0.10^a^	2.23±0.07b^c^	2.37±0.07^b^	2.14±0.03^c^	2.38±0.04^b^
Isoleucine	4.22±0.17^a^	3.39±0.20^b^	3.83±0.04^a^	3.34±0.13^b^	3.90±0.08^a^
Histidine	1.82±0.08^a^	1.74±0.07^ab^	1.62±0.03^b^	1.43±0.03^c^	1.62±0.06^b^
Threonine	4.12±0.07^a^	3.40±0.14^b^	3.71±0.24^ab^	3.36±0.11^b^	3.72±0.09^ab^
Valine	4.20±0.04^a^	3.45±0.12^b^	3.85±0.16^ab^	3.41±0.03^b^	3.82±0.31^ab^
***Non-essential amino acid***					
Arginine	5.59±0.07^a^	4.55±0.23^b^	4.86±0.10^b^	4.47±0.11^b^	4.84±0.11^b^
Alanine	5.82±0.13^a^	5.07±0.23^b^	5.15±0.11^b^	4.92±0.12^b^	5.14±0.17^b^
Taurine	0.50±0.03^b^	0.62±0.05^a^	0.31±0.03^c^	0.46±0.03^b^	0.31±0.02^c^
Tyrosine	3.14±0.01^a^	2.65±0.19^b^	2.77±0.14^b^	2.52±0.05^b^	2.76±0.06^b^
Glycine	7.21±0.05^a^	5.08±0.02^c^	6.41±0.17^b^	6.16±0.12^b^	6.35±0.03^b^
Aspartic acid	10.24±0.32^a^	8.43±0.18^c^	9.34±0.13^b^	8.32±0.13^c^	9.34±0.07^b^
Cysteine	0.92±0.03	0.78±0.05	0.88±0.05	0.79±0.04	0.88±0.02
Serine	3.87±0.21	3.42±0.21	3.62±0.23	3.31±0.14	3.62±0.09
Proline	5.55±0.13^a^	4.16±0.10^b^	3.50±0.02^c^	4.21±0.24^b^	3.50±0.10^c^

Values are expressed as the mean ± SE of three replicate groups. In the same row, means with different subscripts are significantly different (ANOVA and Tukey Post-Hoc Multiple Comparisons Test (P<0.05)).

### Fatty acid composition

The fatty acid (FA) composition (% of total fatty acids) of juvenile barramundi muscles was significantly influenced by the bioprocessed and unprocessed PBM at varying levels (Table 6). The total SFA was lower (P*<*0.05) in 100BPBM when compared to the control and other diets. Among the SFA, palmitic acid (C16:0) and stearic acid (C18:0) were the most abundant of total FAs in all experimental groups, whereas lauric acid (C12:0) and lignoceric acid (C24:0) were the least abundant among the groups. The lowest ΣMUFA values were observed in the control diet and the MUFA levels increased markedly as dietary FM substitution levels increased from 75% to 100% in both bioprocessed and unprocessed PBM diets. In MUFAs, oleic acid (C18:1n-9) was the predominant fatty acid in all groups, with the highest percentage observed in total FM replacement diets of 100PBM and 100BPBM, and the lowest value detected in the control. Total PUFAs decreased significantly in all dietary groups compared to the control and fish fed 100% replacement diets of 100PBM had the lowest levels of n-3 PUFAs. In the n-3 PUFAs, EPA and DHA levels in fish fed the control diet were significantly higher from the rest of the FM protein substituting diets. The highest level of n-6 PUFAs was found in fish fed 100BPBM in which linoleic acid-LA (C18:2n-6) was the predominant FA, representing 13.71% of total FAs in 100BPBM compared to 3.94% in the control.

**Table 6:** Fatty acid profile (% of total fatty acids) in muscles of juvenile barramundi after the completion of feeding trial for 42 days.

	Experimental diets				
	Control	75PBM	75BPBM	100PBM	100BPBM
C12:0	0.14±0.01^a^	0.07±0.01^b^	0.06±0.01^b^	0.08±0.01^b^	0.07±0.01^b^
C13:0	0.04	-	-	-	-
C14:0	1.74±0.10^a^	1.21±0.05^c^	1.24±0.03^c^	1.52±0.06^ab^	1.34±0.12^bc^
C14:1n5	0.02±0.00^c^	0.05±0.00^b^	0.09±0.01^a^	0.10±0.02^a^	0.05±0.01^b^
C15:0	0.51±0.02^a^	0.25±0.01^b^	0.27±0.01^b^	0.29±0.01^b^	0.27±0.01^b^
C15:1	0.06±0.01^a^	0.03±0.00^b^	0.04±0.00^b^	0.06±0.01^a^	0.03±0.01^b^
C16:0	15.54±0.78^bc^	17.99±1.29^ab^	11.99±1.12^d^	20.25±0.68^a^	14.43±0.48^cd^
C16:1n7	2.17±0.21^c^	2.56±0.08^c^	2.70±0.03^b^	3.72±0.08^a^	4.02±0.26^a^
C17:0	0.82±0.02^a^	0.37±0.01^b^	0.42±0.03^b^	0.38±0.02^b^	0.31±0.01^c^
C17:1	0.43±0.04^a^	0.03±0.01^c^	0.30±0.04^b^	0.30±0.02^b^	0.35±0.01^b^
C18:0	9.11±0.77^b^	18.60±2.46^a^	9.13±0.61^b^	9.37±0.63^b^	6.93±0.67^b^
C18:1cis+trans	18.29±1.90^b^	17.81±1.49^b^	29.51±1.17^a^	28.13±1.03^a^	31.57±0.45^a^
C18:2 trans 9	0.25±0.02^d^	0.45±0.02^c^	0.39±0.02^c^	0.55±0.03^b^	1.05±0.01^a^
C18:2n6	3.99±0.26^d^	8.41±0.23^c^	9.41±0.33^c^	11.73±0.30^b^	13.51±0.54^a^
C18:3n6	0.23±0.02^d^	0.40±0.02^c^	0.44±0.01^c^	0.62±0.60^b^	1.15±0.50^a^
C18:3n3	0.59±0.03^e^	0.86±0.02^d^	1.09±0.02^c^	1.43±0.03^b^	1.57±0.02^a^
C18:4n3#	0.40±0.02^b^	0.26±0.01^c^	0.29±0.01^c^	0.35±0.03^b^	0.46±0.02^a^
C20:0	0.33±0.02^a^	0.23±0.01^b^	0.25±0.02^b^	0.26±0.01^b^	0.24±0.02^b^
C20:1	1.83±0.04^a^	1.31±0.02^c^	1.63±0.03^b^	1.80±0.03^c^	1.33±0.03^a^
C20:2	0.27±0.02^ab^	0.23±0.03^b^	0.29±0.01^ab^	0.28±0.02^ab^	0.31±0.01^a^
C21:0	-	0.07	-	-	-
C20:3n6 (DGLA)	0.18±0.01^d^	0.46±0.01^c^	0.47±0.01^c^	0.54±0.02^b^	0.76±0.01^a^
C20:4n6 (ARA)	3.25±0.04^a^	2.93±0.05^b^	3.28±0.10^a^	2.46±0.03^c^	3.23±0.10^a^
C20:3n3	-	-	-	-	-
C22:0	0.02	-	-	-	-
C20:5n3 (EPA)	4.64±0.46^a^	2.96±0.07^cd^	3.53±0.08^bc^	2.78±0.09^cd^	3.72±0.21^b^
C22:1n9	0.21±0.01^a^	0.16±0.01^bc^	0.17±0.01^bc^	0.19±0.01^ab^	0.15±0.01^c^
C22:2	-	-	-	-	-
C23:0	0.04	-	-	-	-
C22:4n6#	2.63±0.25^a^	1.05±0.01^bc^	1.30±0.03^b^	0.63±0.02^d^	0.73±0.02^cd^
C24:0	0.27±0.02^a^	0.07±0.01^ab^	0.07±0.01^ab^	0.07±0.01^ab^	0.13±0.01^ab^
C22:5n3#	2.40±0.12^b^	8.35±2.71^a^	2.66±0.12^b^	2.13±0.05^b^	2.67±0.06^b^
C24:1	0.28±0.02^a^	0.18±0.01^b^	0.16±0.01^bc^	0.172±0.02^b^	0.13±0.02^c^
C22:6n3 (DHA)	29.38±1.74^a^	12.71±0.83^c^	18.83±0.46^b^	9.76±0.22^d^	9.46±0.30^d^
ΣSFA	28.48±1.47^bc^	38.79±3.29^a^	23.43±1.03^c^	32.23±0.71^b^	23.72±1.05^c^
ΣMUFA	23.92±1.67^c^	25.78±0.27^bc^	30.50±0.32^b^	36.35±0.70^a^	35.09±0.75^a^
ΣPUFA	47.96±2.65^a^	38.84±2.50^b^	41.69±0.84^b^	32.99±0.57^c^	38.32±1.18^b^
Σn-3	37.41±2.12^a^	25.15±2.49^b^	26.40±0.54^b^	16.46±0.39^c^	17.89±0.50^c^
Σn-6	10.29±0.56^d^	13.24±0.16^c^	14.90±0.43^b^	15.99±0.40^b^	19.38±0.68^a^
Σn-3/Σn-6	3.64±0.09^a^	1.90±0.23^b^	1.78±0.06^b^	1.03±0.04^c^	0.93±0.01^c^
ΣPUFA/ΣSFA	1.73±0.17^a^	1.11±0.22^b^	1.80±0.09^a^	1.03±0.03^b^	1.65±0.13^a^
IA	0.26±0.02^ab^	0.30±0.02^a^	0.20±0.02^b^	0.31±0.02^a^	0.24±0.02^ab^
IT	0.13±0.01^c^	0.19±0.02^b^	0.13±0.01^c^	0.29±0.01^a^	0.20±0.01^b^
HH	3.69±0.25^bc^	2.90±0.35^cd^	5.37±0.40^a^	2.70±0.11^d^	4.21±0.20^b^

Values are mean ± SE of three replicate fish from each treatment. Values in the same row with different superscript letters (a,b,c) are significantly different based on Tukey's test (One-way ANOVA, P<0.05). ΣSFA = sum of saturated fatty acids; ΣMUFA, sum of monounsaturated fatty acids; ΣPUFA, sum of polyunsaturated fatty acids; Σn-3 PUFA, sum of omega-3 polyunsaturated fatty acids; Σn-6 PUFA, sum of omega-6 polyunsaturated fatty acids; IA, index of atherogenicity; IT, index of thrombogenicity and HH, (hypocholesterolemic/hypercholesterolemic ratio; -, not detected.

### Nutritional quality indices

Lipid nutritional quality indices of fish fed bioprocessed and unprocessed PBM at varying levels are shown in Table 6. The higher value of ΣPUFA/ΣSFA ratio was found in the bioprocessed feeds along with the FM-based control compared to the unprocessed feeds. The Σn-3/Σn-6 ratio was significantly higher (P<0.05) in the control diet and decreased with increasing levels of PBM in the diet with the lowest value recorded in complete FM replacement diets of 100PBM and 100BPBM. Fish fed the bioprocessed feeds of 75BPBM and 100BPBM, and the control showed the highest HH values compared to the unprocessed PBM diets of 75PBM and 100PBM. Fish fed the bioprocessed 100% FM replacement diet of 100BPBM showed significantly lower IA and IT values, and higher HH values when compared to the unprocessed diet of 100PBM. Whereas in the 75% FM replacement level, only the HH index was significantly different between bioprocessed and unprocessed diets. The higher HH value was found in the bioprocessed diet of 75BPBM compared to the unprocessed 75PBM.

### Principal component analysis (PCA)

**Fig 2:**
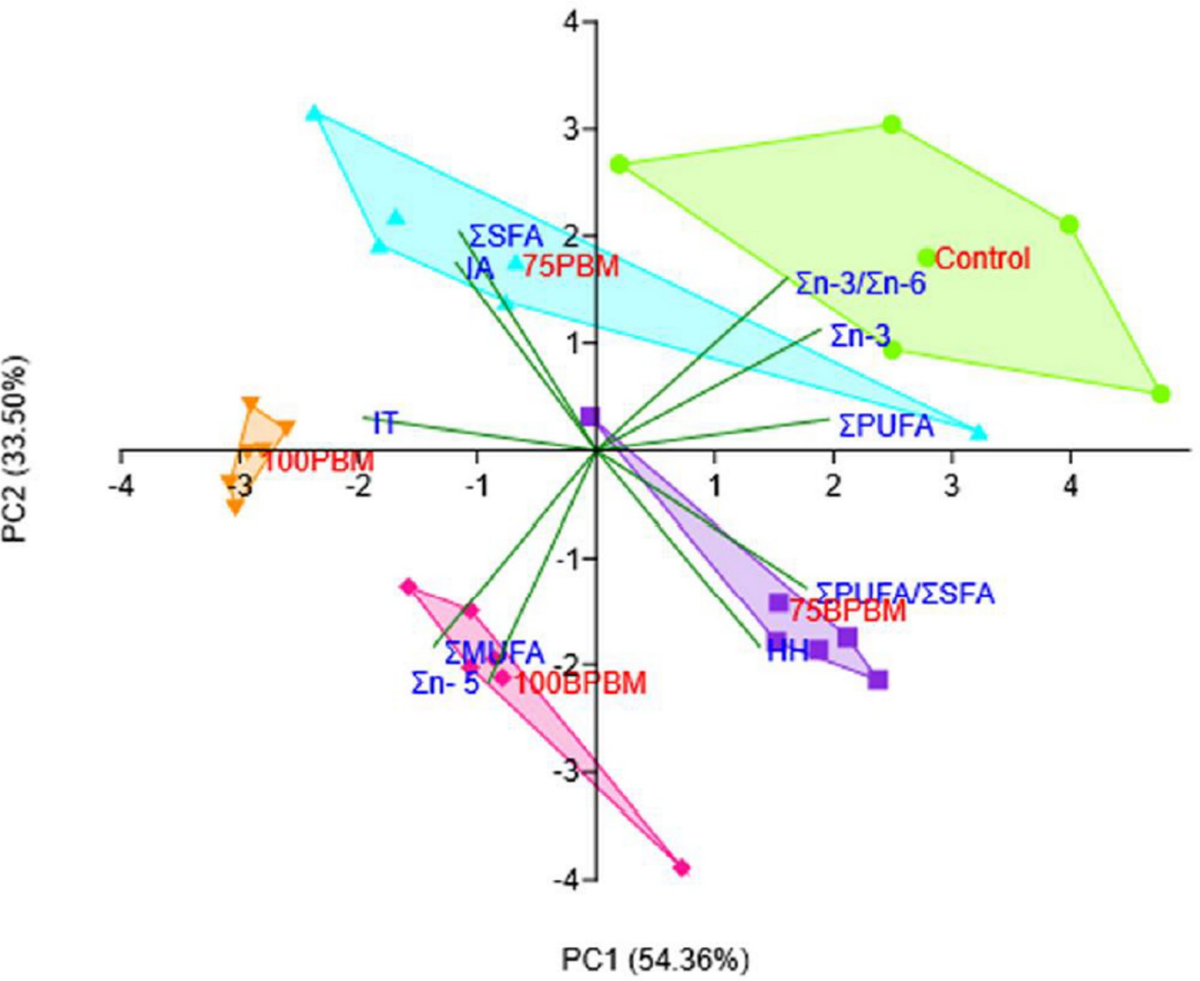
Graphical representation of first two principal components (PC1-PC2) biplot of fatty acid composition in juvenile barramundi fed with different levels of bioprocessed and unprocessed PBM. The figure depicts positive and negative association between variables and clustering between samples based on score obtained from analysis. Different markers represent samples from different dietary treatments used in the analysis and vectors indicate how the variables contributed to the formation of PC1 and PC2.

The most significant principal component (PC1 and PC2) and their statistical loadings (loading values ≥0.30) generated from selected FAs in the muscle tissue of barramundi were considered for the PCA analysis. Two principal components extracted in the current study explained 87.86% of the total variability in the dataset. According to the Kaiser [41] rule, only eigenvalues >1.0 were considered significant elements for data variance. The estimated eigenvalue for the first principal component (*i*.*e*., PC1) was 5.44 and comprised 54.36% of the variance in the dataset, while the second (*i*.*e*., PC2) had an eigenvalue of 3.35, and accounted for 33.35%. The mutual projections of loading vectors using PC1-PC2 are presented in Fig 2 to visualize the specific pattern of correlation between variables. Loading values ≥0.30 were considered significant according to Lombarte, Gordoa [42]. Scores from PC1 indicated that the following FAs: ΣPUFA (0.40), Σn-3 (0.38), Σn-3/Σn-6 (0.33) and ΣPUFA/ ΣSFA (0.36), had a positive contribution while IT (-0.40) influenced PC1 in a negative manner. PC2 showed the highest positive loading for ΣSFA (0.41), Σn-3/Σn-6 (0.33) and IA (0.35), while Σ MUFA (-0.37), Σn-6 (-0.44) and HH (-0.37) influenced PC2 in a negative manner. The PCA analysis demonstrated the clear effects of dietary treatments on the fatty acids profile of juvenile barramundi where the control treated group was discriminated by the Σn-3 and Σn-3/Σn-6 and to a lesser extent by the ΣPUFA. The 75PBM diet was discriminated by an abundance of ΣSFA and IA, while the 75BPBM group was discriminated by an abundance of HH and ΣPUFA/ ΣSFA. 100PBM was mostly characterized by IT whereas 100BPBM treated groups were discriminated from other test groups by the contents of Σn-6 and ΣMUFA.

## Discussion

In the present study, the inclusion of both bioprocessed and unprocessed PBM meal at levels of 75% in practical diets did not reduce growth performance of juvenile barramundi. These results are in agreement with a previous study with humpback grouper, *Cromileptes altivelis*, in which no significant differences in growth parameters were observed when FM was replaced with up to 75% of locally sourced feed-grade PBM [43]. Zhou, Zhao [44] reported replacement of FM with up to 60% of pet food-grade PBM did not decrease growth performance in terms of FBW, WG and FCR in juvenile cobia, *Rachycentron canadum*. In the present study, the total replacement groups of 100PBM and 100BPBM led to a significant decrease of FBW, WG and FCR in juvenile barramundi. The poorer growth performance in complete FM replacement diets of 100PBM and 100BPBM may be associated with the deficiency of some essential amino acids, and variability in biochemical composition, with high levels of ash and low digestibility in these diets [9, 45]. Wolters, Barrows [46] also reported growth reductions in Atlantic salmon, *Salmo salar* due to increased accumulation of nitrogenous wastes. However, one of our previous study has found that up to 100% FM from a control diet was replaced by a combination of PBM and tuna hydrolysate meal, without altering growth performance of the juvenile barramundi [47].

The VSI and CF in the present study were not significantly influenced by the replacement of FM by PBM at various levels. These results were in agreement with Zhou, Zhao [44] for juvenile cobia, *Rachycentron canadum* and Shapawi, Ng [43] for the humpback grouper, *Cromileptes altivelis*. The HSI showed a significant increase in fish fed 100BPBM and 100PBM compared to remaining diets, the results in agreement with previous studies reporting an increase in HSI with the increasing inclusion of PBM in diets as noted in previous studies [48–50]. The increased HSI with fish fed 100PBM and 100BPBM may be due to increased lipid deposition in the liver, resulting in hepatic alterations, including hepatic steatosis. The similar effects were reported in a study on juvenile barramundi when FM was replaced at 75% with tuna hydrolysate [32]. However, Shapawi, Ng [43] and Hu, Wang [51] reported no significant variation in HSI even up to 100% replacement of FM with PBM in humpback grouper, *Cromileptes altivelis* and gibel carp, *Carassius auratus gibelio*, respectively.

The ingredients in fish feed has a marked effect on gut morphology as well as the overall health condition of fish [52, 53]. Longer fold and villus height of gut are associated with good health and the high absorptive efficiency of the diet, whereas shorter fold and villus height are indications of poor nutrient utilization and absorption, reduced immune functions, and subsequent lower growth performance of fish [54]. The SEM images from this study revealed that fish fed the 75BPBM had higher microvilli density when compared to the control and the rest of the dietary groups. This could possibly be due to the effect of fermentation of the diet with probiotic bacteria *Lactobaccilus casei*, resulting in a superior beneficial effect than when PBM was used alone. This result is in agreement with Wang, Zhou [55] who reported improved gut morphology (intestinal folds, enterocytes, and microvilli) of juvenile turbot, *Scophthalmus maximus*, fed soybean meal fermented with *Lactobacillus plantarum* when compared to a non-fermented diet. The significantly lower microvillus density in fish fed the 100PBM and 100BPBM diets may be attributed to the deleterious effect of sub-optimal feed on digestion and absorption of fish.

Both bioprocessed and unprocessed PBM diets had no significant effect in fish muscles moisture, protein, lipid and ash content of juvenile barramundi in the present study. This observation was in agreement with several studies demonstrated no effects of dietary PBM on the whole-body composition of fish [50, 56]. However, Shapawi, Ng [43] who reported that whole-body protein content was significantly lower in humpback grouper fed 100% replacement diet with PBM. In contrast, Yang, Xie [48] reported significantly higher whole-body protein with increasing dietary PBM in gibel carp, *Carassius auratus gibelio*. Such changes in whole-body composition are likely to be associated with the species specific differences, compounding dietary and environmental factors, associated with the varying levels and quality of protein in PBM relative to FM [43]. The amino acids (AAs) in barramundi muscles in this study were similar as reported by Riche (2015). Almost all the AAs (essential and non-essential) were depleted by the substitution of FM by PBM in the diets of barramundi which reflected in the test diets. Some AAs, including Arginine, Methionine, Glutamic acid, Alanine, Glycine and Proline were higher in PBM than fishmeal, but AAs in fish muscles were higher in fish fed control diet than the fish fed different levels of bioprocessed and unprocessed PBM suggesting that these AAs were not well utilized by the fish. In contrast, the Lysine in control diet was higher, but it was not different between control and bioprocessed PBM fed fish muscles.

Substitution of FM by PBM modified the fatty acid composition in the muscle tissue of fish. In the present study, FA analyses in the experimental diets showed a lower level of Σn-3 PUFA (notably 22:5n-3 and 22:6n-3), along with a higher volume of Σn-6 PUFA (notably 18:2n-6) when compared to the FM. This trend was reflected in the Σn-3 PUFA and Σn-6 PUFA contents of the fish muscles after 42-days of experimentation. Furthermore, the dietary n-3 PUFAs normally decrease and n-6 PUFA subsequently increase with the increasing substitution levels of FM protein by terrestrial animal derived protein, which are often deficient in n-3 PUFAs [17]. In the present study, fish receiving 100PBM and 100BPBM diets had lower Σn-3 PUFA and higher Σn-6 PUFA in the fish muscles which aligns with the findings in fry of Nile tilapia, *Oreochromis niloticus*, fed 100% PBM protein-based diets [57]. However, an exception was observed for Σn-3 PUFA in fish fed 75BPBM, which was not lessened compared to the control, as the supplied 75BPBM diet possessed a higher EPA and DHA compared to the unprocessed one in the 75PBM diet. Human epidemiological studies have implicated that myristic acid (C14:0) positively correlated with higher cholesterol levels in plasma which increased the risk of cardiovascular disease [58, 59]. Therefore, lower amounts of myristic acid in food may be beneficial for human health [58]. In the present study, the lowest amount of myristic acid (14:0) was found in the diets of 75PBM, 75FPBM and 100FPBM compared to the FM-based control diet.

According to Ulbricht & Southgate [39], the indices of atherogenicity (IA) and thrombogenicity (IT) of a diet in humans indicate a state of coronary heart disease due to the obstruction of coronary vessels by atherosclerosis or thrombosis in the circulatory system. Therefore, the lower the indices of IA and IT values of a diet, the higher the protection for cardiovascular disorders. On the other hand, the HH ratio refers to the proportion of **Σ**hypercholesterolemic FA/**Σ**hypercholesterolemic FA and this value is related to cholesterol metabolism. In contrast to IA and IT, the higher HH values are more beneficial for human welfare. In this study, the lowest IT values were observed in 75BPBM and control diets compared to rest of the diets whereas the highest HH values were observed in bioprocessed diets of 75BPBM and 100BPBM compared to the remaining diets. The lower IT values and higher HH values found in these groups might be due to the higher levels of PUFA present in diets supplied by these fish groups. Approximately 2% lipid reduction in BPBM (11.70%) than PBM (13.50% lipid) may have a greater influence on improved nutrition and flesh quality of fish fed bioprocessed diets. Considering the lipid nutritional indices, the consumption of these species fed with bioprocessed as well as FM-based controlled diets may be beneficial to human health when compared to fish cultured with unprocessed PBM diets.

The PCA analysis was applied to test the influences of the different dietary inclusions of bioprocessed and unprocessed PBM on the FA profiles of fish and also indicating suitable FAs for human consumption. The loading plot of PC1-PC2 was explanatory to 87.86% of the total variation and showed a clear differentiation of samples based on their dietary inclusion levels of PBM. In the PCA biplot and convex hulls, fish fed control and 75BPBM were clustered in the positive site of PC1 with Σn-3, Σn-3/Σn-6, HH and ΣPUFA/ ΣSFA. These groups were separated from 75PBM, 100PBM and 100BPBM treated groups which clustered in the negative region of PC1 with IA, IT, ΣMUFA and Σn-6. The position of these factors and variables in the plot revealed that the 75BPBM diet positively influenced the HH and ΣPUFA/ΣSFA while 75PBM, 100PBM and 100BPBM had a negative correlation with IA and IT.

## Conclusions

The results obtained in this study demonstrated that 75% FM replacement diets (both bioprocessed and unprocessed PBM) did not impair growth performance, gut morphology and liver health of juvenile barramundi. The nutritional quality indices of the fish muscle (IA, IT and HH) were improved with bioprocessed PBM diets compared to unprocessed PBM diets. These results may indicate that consumption of fish cultured in bioprocessed diets, and hence with different fatty acid profiles, may generate some human health benefits, potentially relating to reducing the risks of cardiovascular disease. However, further work, including clinical trials, would be required to fully determine a human health outcome.
